# An invasive legume increases perennial grass biomass: An indirect pathway for plant community change

**DOI:** 10.1371/journal.pone.0211295

**Published:** 2019-01-25

**Authors:** Jennifer M. Fill, Eleanor Pearson, Tiffany M. Knight, Raelene M. Crandall

**Affiliations:** 1 School of Forest Resources and Conservation, University of Florida, Gainesville, Florida, United States of America; 2 Department of Biology, Washington University in St. Louis, St. Louis, Missouri, United States of America; 3 Department of Community Ecology, Helmholtz Centre for Environmental Research- UFZ, Halle (Saale), Germany; 4 Institute of Biology, Martin Luther University Halle-Wittenberg, Halle (Saale), Germany; 5 German Centre for Integrative Biodiversity Research (iDiv) Halle-Jena-Leipzig, Leipzig, Germany; Eidgenossische Forschungsanstalt fur Wald Schnee und Landschaft Institut fur Schnee- und Lawinenforschung, SWITZERLAND

## Abstract

The presence of native grasses in communities can suppress native forbs through competition and indirectly benefit these forbs by suppressing the invasion of highly competitive exotic species. We conducted a greenhouse experiment to examine the potential of direct and indirect interactions to influence the aboveground biomass of four native forb species in the presence of the native perennial grass *Schizachyrium scoparium* and exotic invasive *Lespedeza cuneata*. We examined patterns of growth for the invasive legume, the perennial grass, and four native species in four scenarios: 1) native species grown with the grass, 2) native species grown with the legume, 3) native species grown with both the grass and legume together, and 4) native species grown alone. *Schizachyrium scoparium* significantly decreased biomass of all forb species (*p*<0.05). In contrast, *L*. *cuneata* alone only significantly affected biomass of *Asclepias tuberosa; L*. *cuneata* increased the biomass of *A*. *tuberosa* only when the grass was present. When *S*. *scoparium* and *L*. *cuneata* were grown together, *L*. *cuneata* had significantly lower biomass (*p* = 0.007) and *S*. *scoparium* had significantly greater biomass (*p* = 0.002) than when each grew alone. These reciprocal effects suggest a potential pathway by which *L*. *cuneata* could alter forb diversity in grassland communities In this scenario, *L*. *cuneata* facilitates grass growth and competition with other natives. Our results emphasize the importance of monitoring interactions between exotic invasive plant species and dominant native species in grassland communities to understand pathways of plant community change.

## Introduction

Native perennial grasses are a keystone functional group of grassland and savanna communities. They influence fundamental ecosystem processes such as fire regimes and nutrient cycling [1–3] but they also have been shown to increase ecosystem resilience and community resistance to invasion [4–6]. As long-lived individuals that achieve high biomass, native perennial grasses may be particularly influential in competing with other plant species and in determining community invasion dynamics over the long term [7].

Several studies have experimentally demonstrated that native grasses are important in community resistance to invasion. For example, in Kansas tallgrass prairie, native perennial grass biomass had a negative effect on the abundance and richness of exotic plant species after fifteen years [8]. In Wyoming sagebrush-bluebunch wheatgrass communities, perennial grass removal nearly doubled the density and biomass of an invasive grass [9]. Evidence therefore suggests that established perennial grasses are strong competitors and should be considered a focus in perennial grassland restoration efforts [10–11], particularly where remnant grasses are still present [12].

Perennial grasses and invasive plants also affect co-occurring native species’ population dynamics. At intermediate densities, established perennial grasses can increase native plant diversity [5], such as by acting as nurse plants [13], while at high densities they tend to suppress native plant growth by limiting space and resource availability [14]. In a shortgrass steppe community, removal of a dominant native perennial grass resulted in increased densities of native species [15]. Similarly, exotic invasive plants are notorious for their suppression of native plant communities, and their presence has been shown to reduce native species growth, survival, and fecundity [16–17] and native diversity [18–22]. The presence of perennial grasses is expected to have positive effects on native species through their suppression of exotic invasive plants and negative effects through their own direct competition.

Invasive plants, however, can enhance native species’ survival and growth through mechanisms such as habitat modification or competitive release [23]. For example, the biomass of a native perennial grass in Argentina was greater when it was growing near the invasive legume *Lotus tenuis* (narrowleaf trefoil), presumably due to increased nitrogen levels [24]. Non-native species can serve as nurse plants for regenerating native seedlings by ameliorating otherwise stressful environmental conditions [25–26]. The potential for such facilitative interactions should not be overlooked [27]; both negative and positive interactions among native and invasive species can generate cascading effects on higher trophic levels [23]. We conducted a greenhouse experiment to test the effects of the presence of an exotic invasive legume and an established perennial grass on the aboveground biomass of native forbs, and to examine the reciprocal effects of the invasive legume and perennial grass on each other’s biomass. We examined patterns of growth for the invasive legume, the perennial grass, and four native species in four scenarios: 1) native species were grown with an established native perennial grass, 2) native species were grown with an invasive legume, 3) native species were grown with both the established grass and invasive legume together, and 4) native species were grown alone. We predicted that when grown alone with native species, the dominant perennial grass would increase biomass of co-occurring natives during their early recruitment. We further predicted that when grown alone with native species, the invasive legume should decrease native species’ growth. In pots with both the perennial grass and invasive legume, we predicted that the grass would decrease the growth of the invasive, indirectly resulting in greater growth of co-occurring natives.

## Materials and methods

### Study species

*Lespedeza cuneata* (sericea lespedeza) is a legume that invades open roadsides, prairies, and old fields in the Midwestern U.S.A. It was introduced from Asia in the early 1900s primarily to control roadside erosion and provide forage and has since widely invaded pasture and grasslands across the U.S.A. from Kansas to the east coast [28]. *Lespedeza cuneata* has been shown to directly suppress native forbs by shading them out early in grassland restoration [29]. Reclamation studies also suggest *L*. *cuneata* inhibits natural seedling establishment [30].

The native species chosen for this study are typically dominant in remnant prairies and thus often used for prairie restoration [31]. The four focal native species are *Monarda fistulosa* (wild bergamot), *Coreopsis lanceolata* (lanceleaf tickseed), *Asclepias tuberosa* (butterfly milkweed), and *Chamaecrista fasiculata* (partridge pea). These species relatively common, although given the high diversity in native prairies, one forb species is rarely dominant. All are herbaceous, perennial plants with high germination rates and short time to reproduction. The native grass is *Schizachyrium scoparium* (little bluestem), a perennial, upright bunchgrass which is ubiquitous in Midwestern U.S.A. prairies [32]. *Schizachyrium scoparium* was historically one of the dominant grasses of the midwestern tallgrass prairie region and grows in a variety of environments from moist to dry soils [32].

### Greenhouse experiment

Seeds of *L*. *cuneata* were collected from reproducing plants during November 2013, stored at 3°C for five months, and scarified prior to planting. Native seeds and 2-year old *S*. *scoparium* rhizomes (the “established grass” in our experiment) were purchased from Prairie Moon Nursery (https://www.prairiemoon.com/) and Missouri Wildflowers Nursery (http://mowildflowers.net/), respectively. Both companies specialize in native plants for prairie restoration.

During May, rhizomes were planted and seeds were sowed in 5-gallon pots containing Metro-Mix 360. This soil, a combination of Canadian sphagnum peat moss, bark, vermiculite and dolomitic limestone, which has high water retention necessary for germination and seedling success. We set up between four and six replicates of four treatments, haphazardly arranged in five rows to account for spatial variability in greenhouse conditions. The treatments were: 1) grass rhizome and native species; 2) the invasive legume and native species; 3) grass rhizome, the invasive legume, and native species; 4) control (four focal native species). In treatments with *S*. *scoparium*, grass rhizomes were planted in the center of each pot. In treatments without *S*. *scoparium*, a segment of round PVC 5cm in diameter that extended from bottom of pot to soil surface was inserted in the center of pots to take up approximately the same space as the rhizomes, which have been shown to grow to deep depths in grassland systems [33]. Thus, competition for space should not differ significantly between pots. For each treatment, we planted 20 seeds of the forb species around the rhizome or PVC. In treatments with *L*. *cuneata*, we sowed four seeds of the legume and of each native species; in treatments without *L*. *cuneata*, we sowed five seeds each native species. The extra individual of each of the four native forbs in treatments without *L*. *cuneata* could potentially affect experimental outcomes (e.g., via increased intraspecific competition). However, it is much more likely that overall density (i.e., of all species) at a community level would affect outcomes. We controlled for the density of individuals in our experimental design which ultimately resulted from differences in survival between pots (see *Statistical analyses*).

Plants were grown in the greenhouse at Washington University in St. Louis for 14 weeks. Pots were watered daily with 1500 mL of water and ladybugs were released on plants, as needed, to control aphids. During this time, there were some reproducing individuals of three species: *S*. *scoparium*, *A*. *tuberosa*, and *C*. *fasiculata*. At 14 weeks, individual plants were harvested. Plants were clipped at soil level, dried in an oven for 48 hours or until completely dry at 40 °C, and weighed to quantify aboveground biomass for each individual (see S1 Appendix for sample sizes). It was not possible to quantify belowground biomass because the roots of all species were so entangled that we could not separate them and differentiate between species.

### Statistical analyses

We ran all analyses in R 3.4.3, R Core Development Team, 2017. We conducted four two-way ANOVAs to examine the effects of *L*. *cuneata* and *S*. *scoparium* on the aboveground biomass of each of the four forb species. Our main factors were the independent and interactive effects of grass and invasive presence/absence. We included the number of forb individuals (excluding the grass) that survived in each pot as a random effect (plant density) to control for differences in the number of individuals per pot. We conducted two Welch’s two-sample t-tests to compare *S*. *scoparium* aboveground biomass between pots with and without *L*. *cuneata*, and to compare *L*. *cuneata* aboveground biomass between pots with and without *S*. *scoparium*.

## Results

**Fig 1:**
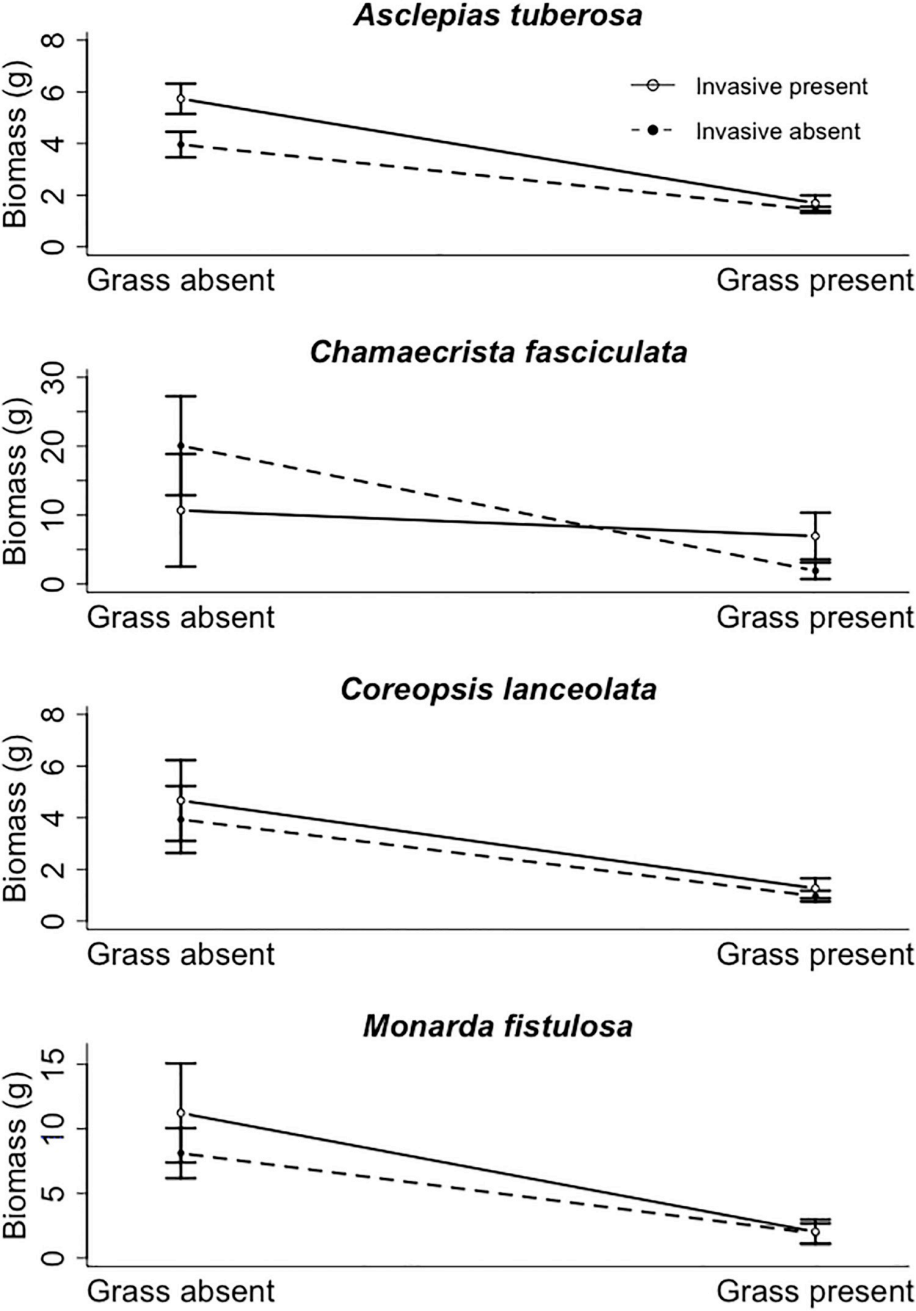
Experimental biomass results for forb species. Aboveground biomass (mean ± standard error) for four forb species grown in pots with and without the native grass, *Schizachyrium scoparium*, and the invasive legume, *Lespedeza cuneata*.

Native perennial grass and invasive legume presence had different effects on the growth of native grassland forbs. The perennial grass alone significantly decreased the aboveground biomass of all native forbs (Table 1; Fig 1). In contrast, the invasive legume alone only significantly affected biomass of *A*. *tuberosa* (Table 1). When the grass was absent, *A*. *tuberosa* biomass was higher when growing with the invasive (Fig 1). When the grass was present, *A*. *tuberosa* biomass was not affected by the presence or absence of the invasive (Fig 1). None of the forb species were significantly affected by plant density (Table 1).

**Table 1:** ANOVA table for tests of treatment and plant density effects on forb aboveground biomass.

		*Asclepias tuberosa*			*Coreopsis lanceolata*			*Chamaecrista fasiculata*			*Monarda fistulosa*		
	df	MS	*F*	*P*	MS	*F*	*P*	MS	*F*	*P*	MS	*F*	*P*
Grass	1	171.12	63.21	<0.001*	81.93	9.73	0.004*	1228.20	4.61	0.044*	427.10	11.27	0.002*
Invasive	1	17.13	6.33	0.014*	1.64	0.20	0.662	32.40	0.12	0.731	22.40	0.59	0.448
Grass x Invasive	1	14.17	5.23	0.025*	1.41	0.17	0.685	226.7	0.852	0.367	14.7	0.39	0.539
Plant density	1	2.58	0.95	0.333	8.35	0.99	0.327	137.4	0.516	0.481	2.80	0.07	0.787
Error		2.71 (66)			8.42 (30)			266.2 (21)			37.9 (29)		

Results are shown for aboveground biomass of forb species grown in pots with a perennial grass (*Schizachyrium scoparium*) and invasive legume (*Lespedeza cuneata*). Asterisks indicate significant effects. The degrees of freedom for the error term are in parentheses after the mean square (MS) value. Because the number of surviving individuals varied among species, the error degrees of freedom also differed

**Fig 2:**
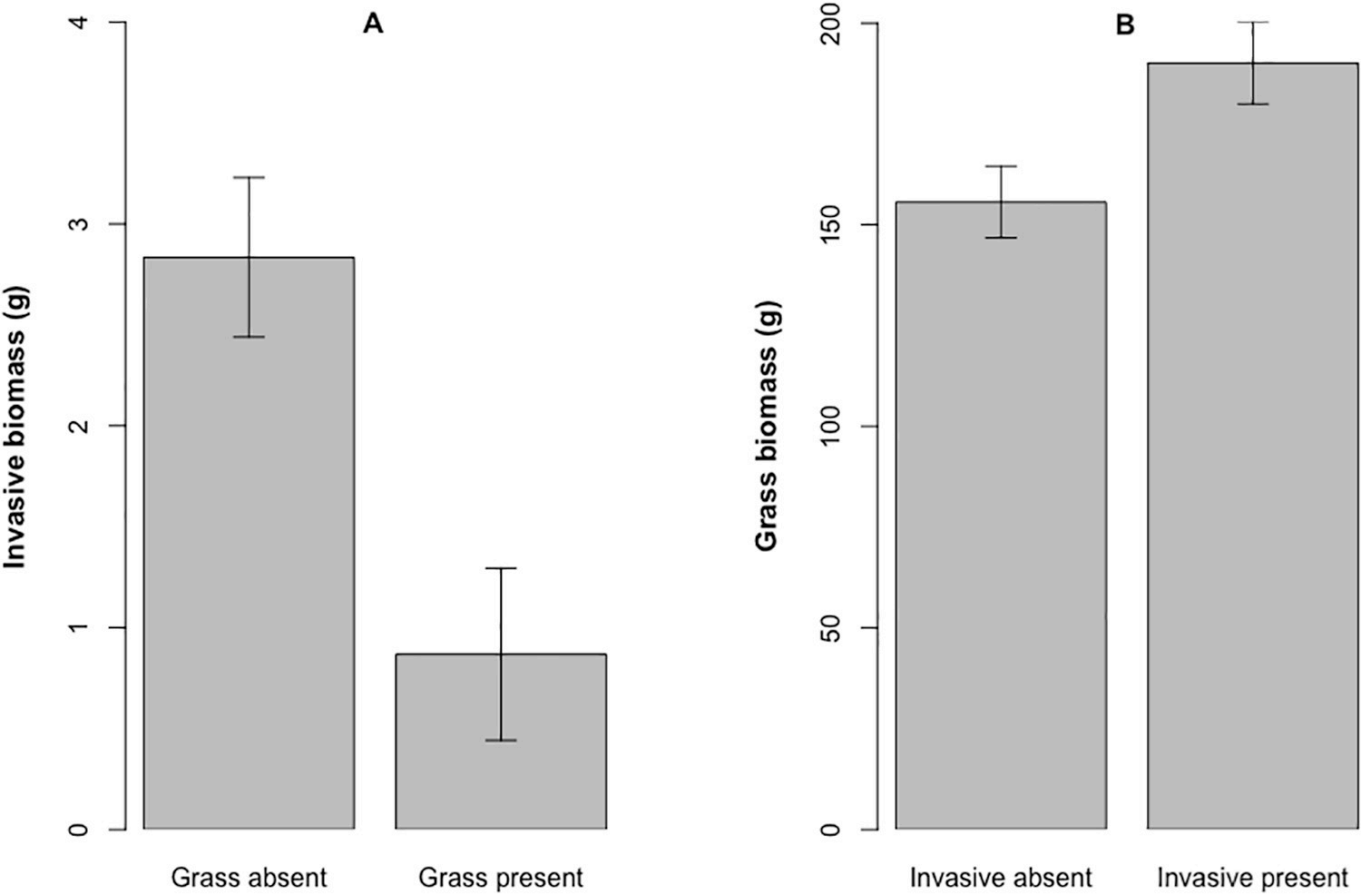
Experimental biomass results for the grass and legume. **(a)** Aboveground biomass of invasive *Lespedeza cuneata* when grown in pots with and without the native grass, *Schizachyrium scoparium* (*P* = 0.007) **(b)** Aboveground biomass of *Schizachyrium scoparium* when grown in pots with and without *Lespedeza cuneata* (*P* = 0.002). In all pots, four species of native, perennial forbs were present.

The native grass and invasive legume each had different effects on each other’s growth. When *L*. *cuneata* grew with *S*. *scoparium*, the aboveground biomass of *L*. *cuneata* was significantly lower relative to pots in which *L*. *cuneata* grew with only native species (Fig 2A). In contrast, *S*. *scoparium* had significantly greater biomass when grown with *L cuneata* plus the native species than when grown with only the native species (Fig 2B).

## Discussion

When grown separately, the native perennial grass, but not the invasive legume, suppressed native species’ aboveground biomass. Native forbs grown with *S*. *scoparium* had lower biomass than those grown alone, but counter to our hypothesis, native forbs were generally not affected by the invasive legume. The exception to this result was *Asclepias tuberosa*, which had more biomass where *L*. *cuneata* was present and the grass was absent. Although light limitation is a mechanism by which both *L*. *cuneata* and perennial grasses can negatively affect co-occurring native species [29, 34], this type of competition was unlikely in our study. The four native forbs are relatively tall-statured species that quickly exceeded the height of *S*. *scoparium*. It is possible that belowground interactions have a greater effect on growth. *Lespedeza cuneata* is a nitrogen-fixer and increased nitrogen in pots with *L*. *cuneata* might have ameliorated any negative direct effects of competition between *L*. *cuneata* and natives, and even facilitated growth (e.g., *A*. *tuberosa*). *Lespedeza cuneata* is also known to alter soil bacterial and fungal community composition [35, 36] which may have differentially influenced forb growth.

Although the perennial grass did reduce the growth of *L*. *cuneata* as we hypothesized, it was surprising that *L*. *cuneata* significantly increased perennial grass aboveground biomass in our study. [36] also found that *Sorghastrum nutans* biomass was higher when grown with *L*. *cuneata* than with a conspecific. Conversely, [37] found that the native grass *Panicum virgatum* had reduced growth in soil conditioned by *L*. *cuneata*, which has been shown to disrupt the mychorrhizal fungal communities associated with *P*. *virgatum* [38]. These outcomes suggest that nutrient levels or microbial communities could also be mediating grass-legume interactions. *Schizachyrium scoparium* has shown an ability to take advantage of nutrient pulses [39] and may have therefore gained a competitive edge over the invasive due to increased nitrogen levels. *Lespedeza cuneata* might also have disrupted soil microbial communities in a manner favorable to the perennial grass.

**Fig 3:**
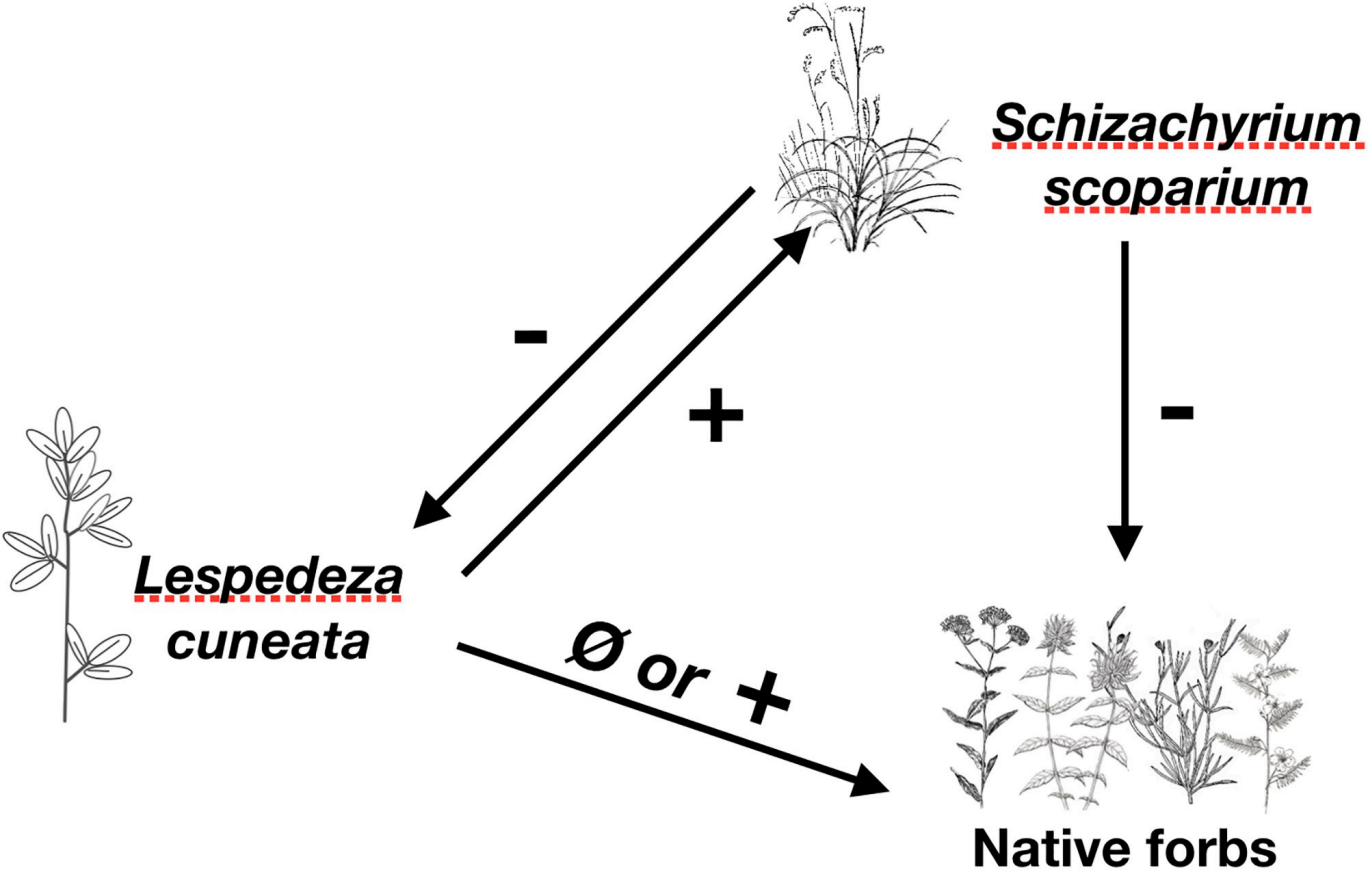
Conceptual diagram of experimental outcomes. Arrows and signs show interactions among an invasive legume (*Lespedeza cuneata*), perennial grass (*Schizachyrium scoparium*) and co-occurring native perennial forbs.

Our results suggest a potential pathway by which *L*. *cuneata* could alter biodiversity in grassland communities. Given *S*. *scoparium’s* negative effect on forb aboveground biomass, we suggest that *L*. *cuneata* facilitates competition of *S*. *scoparium* with co-occurring natives (Fig 3). In this scenario (Fig 3), *Lespedeza cuneata* initially establishes in an area of low bunchgrass density. By increasing nitrogen levels or altering microbial communities, it increases grass growth, thereby enhancing the competitive advantage of the grass over native forbs. Despite experiencing suppression from the grass, if *L*. *cuneata* persists until high grass density limits grass growth [40] it could have a dominant effect on grassland communities.

There are many mechanisms that could affect the ability of *L*. *cuneata* to eventually become dominant in the community besides the interactions that we investigated here. Initial interactions between the grass and legume could change over time. Although initial interactions might be dominated by the nitrogen supply to the grass, after the legume has increased in biomass and density it might compete the grass for other resources (e.g., water or sunlight; [41]). *Lespedeza cuneata* might also be developing feedbacks with soil microbial communities that would eventually favor its own growth [42] and generate other mechanisms for community change. Alternatively, ecological processes could limit *L*. *cuneata* invasion. Prolific seed production is an attribute that increases *L*. *cuneata*’s invasiveness [43–44]. *Lespedeza cuneata* adults are susceptible to herbivory and fire [45–46]; disturbances such as herbivory or grazing and fire could limit the species’ population growth via effects on seed production.

Our results emphasize the importance of considering interactions between exotic invasive plant species and dominant native grasses to understand pathways of native plant suppression. An invasive plant might appear to have no direct effect on biodiversity, but when interactions with the dominant native grasses are involved, the results become more complex. Studies of interactions among invasive and dominant native species should prevent unexpected outcomes in invasive species management and promote more appropriate and effective management strategies for biodiversity.

## Supporting information

S1 Appendix Biomass data used for analysis. (CSV)
